# Polymorphism of the catechol-O-methyltransferase gene in Han Chinese patients with psoriasis vulgaris

**DOI:** 10.1590/S1415-47572009005000002

**Published:** 2009-01-10

**Authors:** Lin Gao, Hong Li, Kai Li, Zhu Shen, Ling Liu, Chunying Li, Zhengdong Zhang, Yufeng Liu

**Affiliations:** 1 Department of Dermatology, Xijing Hospital, Fourth Military Medical University, ShaanxiChina; 2 Department of Molecular Genetic Toxicology, Nanjing Medical University, NanjingChina

**Keywords:** COMT, gene polymorphism, genetic susceptibility, psoriasis

## Abstract

Psoriasis vulgaris is defined by a series of linked cellular changes in the skin: hyperplasia of epidermal keratinocytes, vascular hyperplasia and ectasia, and infiltration of T lymphocytes, neutrophils and other types of leukocytes in the affected skin. Catechol-O-methyltransferase (*COMT*) *158* polymorphism can reduce the activity of the COMT enzyme that may trigger defective differentiation of keratinocytes in psoriasis. Immunocytes can degrade and inactivate catecholamines via monamine oxidase (MAO) and COMT in the cells. We hypothesized that the *COMT-158G* > *A* polymorphism was associated with the risk of psoriasis vulgaris in Han Chinese people. In a hospital-based case-control study, 524 patients with psoriasis vulgaris and 549 psoriasis-free controls were studied. *COMT-158 G* > *A* polymorphism was genotyped using the PCR sequence-specific primer (PCR-SSP) technique. We found no statistically significant association between the *COMT-158* allele *A* and the risk of psoriasis vulgaris (p = 0.739 adjusted OR = 1.03; 95% CI = 0.81-1.31). This suggests that the *COMT-158 G* > *A* polymorphism may not contribute to the etiology of psoriasis vulgaris in the Han Chinese population.

Psoriasis is a common skin disorder characterized by the focal formation of inflamed, raised plaques that constantly shed scales derived from excessive growth of epithelial cells. The disease is defined by a series of linked cellular changes in the skin: hyperplasia of epidermal keratinocytes, vascular hyperplasia and ectasia and infiltration of T lymphocytes, neutrophils, and other types of leukocytes in the affected skin (Weinstein and Frost, 1968; Simonart and Heenen, 2007). The global incidence of the disease is 1%-3% (Farber and Nall, 1974). Psoriasis is clinically divided into type 1 (onset before 40 years of age) and type 2 (onset after 40 years of age) (Henseler and Christophers, 1985; Ferrandiz *et al.*, 2002).

Psoriasis is a T-cell mediated autoimmune process. Activated lymphocytes, other immune accessory cells and lymphokines have been detected in psoriatic plaques (Dereure and Guilhou, 2003; Piruzian and Abdeev, 2006). Several studies have suggested that T cells play a crucial role in the pathogenesis of psoriasis (Bos *et al.*, 1983; Schlaak *et al.*, 1994; Boehncke *et al.*, 1996; Wrone-Smith and Nickoloff, 1996; Gilhar *et al.*, 1997; Yamamoto *et al.*, 1998; Bos and De Rie, 1999). T-cells accumulate early in psoriatic plaques, and their cytokines induce abnormal keratinocyte proliferation. Catecholamine (CA) can also regulate immune functions. As early as in 1983, Bidart *et al.* found that human T and B lymphocytes had catechol-O-methyltransferase (COMT) immunoreactivity. Later, Balsa *et al.* (1989) reported that monoamine oxide activity (MOA) was present in human blood lymphocytes and granulocytes. Treatment of lymphocytes with pargyline resulted in an increase of intracellular CAs and a decrease of intracellular CA metabolites in the lymphocytes (Karayiorgou *et al.*, 1997; Marino *et al.*, 1999). CA metabolites can be detected in immune cells (Yamamoto *et al.*, 1998; Cosentino *et al.*, 2000). These results imply that immunocytes not only synthesize CAs, but also degrade and inactivate CAs via MAO and COMT in the cells. Previous studies have found that epidermal homogenates from lesional psoriatic skin express higher levels of COMT activity than those from normal skin. This could be due to a high epidermal cell turnover or to an inborn error in the activity of the enzyme (Bamshad *et al.*, 1970).

Although the pathogenesis of psoriasis is still unclear, many studies suggest that the disease has an exceedingly complex genetic basis (Liu *et al.*, 2007). Dimon-Gadal *et al.* (2000) found that oxidative damage occurred before the appearance of typical psoriatic plaques, suggesting that such damage is independent of and may present earlier than the inflammatory infiltration. It has been proposed that oxidative stress of non-differentiated keratinocytes triggers the formation of a defective horny layer (Shilov and Sergienko, 2000).

Catechol-O-methyltransferase (COMT) is an enzyme that catalyses the O-methylation of biologically active toxic catechols and plays an important role in the metabolism of drugs and neurotransmitters (Tursen *et al.*, 2002). In humans, the COMT protein exists as two length variants, soluble (S-COMT) and membrane-bound (MB-COMT), which are encoded by a single gene localized at chromosome 22q11.1-q11.2 (Grossman *et al.*, 1992; Lundstrom *et al.*, 1995; Karayiorgou *et al.*, 1997). The MB-COMT variant has an additional 50 amino acids at the N-terminal, but is otherwise identical to S-COMT (Lundstrom *et al.*, 1991). A single base-pair change (G > A) in exon 4 of the *COMT* gene, resulting in an amino acid change (Val > Met) at codon 158 of MB-COMT and codon 108 of S-COMT, reduces the thermostability and the activity of the enzyme (Lotta *et al.*, 1995; Lachman *et al.*, 1996; Tursen *et al.*, 2002). The two alleles refer to COMT*H, the site-absent (G; Val) allele that encodes the thermostable, high-activity enzyme, and COMT*L, the site-present (A; Met) allele that encodes the thermolabile, low-activity enzyme. The allelic variant only affects the enzyme activity (Palmatier *et al.*, 1999). A common single-nucleotide polymorphism (SNP) in codon 158 of the *COMT* gene (*COMT-158 G* > *A*, rs4680) codes for a substitution of valine (Val) by methionine (Met), resulting in the reduced thermostability and activity of the enzyme (Bertocci *et al.*, 1991; Grossman *et al.*, 1992; Lundstrom *et al.*, 1995; Karayiorgou *et al.*, 1997).

Erdal *et al.* (2004), based on a case-control analysis of a Turkish population, observed that the *COMT*-*158 G* > *A* polymorphism was significantly associated with genotype *COMT AA* and psoriasis cases. They speculated that low enzyme activity could be unable to prevent the formation of toxic o-quinones in psoriatics, and this oxidative stress of keratinocytes could trigger defective differentiation in psoriasis. No differences have been found in COMT polymorphism between psoriatics and control subjects, but the COMT-LL genotype was found significantly increased in the psoriasis patients (Karayiorgou *et al.*, 1997). COMT is important in preventing the formation of toxic o-quinones during epidermal cell synthesis. Therefore, COMT also plays an important regulatory role in the oxidative damage of keratinocytes. Immunocytes can degrade and inactivate CAs via MAO and COMT in the cells (Qiu *et al.*, 2005).

Based on this concept, we genotyped the *COMT*-*158 A* > *G* polymorphism in an ongoing hospital-based case-control study of psoriasis vulgaris in a Han Chinese population. The study sample consisted of 524 patients, 478 with type 1 and 46 with type 2 psoriasis vulgaris, and 549 control subjects recruited from the Xijing Hospital, Fourth Military Medical University, between August and December, 2005. All cases were surveyed, but only Han Chinese (more than 90% of the Chinese population) patients and control subjects were included in the analysis, because genotype frequencies can vary among ethnic groups. The psoriasis-free control subjects were persons who came to the hospital for health examinations and did not have individual or family histories of psoriasis. All cases and controls were examined and diagnosed by dermatologists. We used a questionnaire to collect demographic and other information [stage, type, age of onset, psoriasis area and severity index (PASI) scores], and matched the controls to the cases by age (±10 years) and gender. The response rates of patients and controls approached for participation were both over 90%.

All subjects signed informed consent forms, and donated 5 mL venous blood, used for genomic DNA extraction. This research protocol was approved by the ethics review board of the Fourth Military Medical University.

Venous blood collected from each subject was added to tubes containing 50 mmol EDTA/L, and genomic DNA was isolated from 1 mL anti-coagulated peripheral blood leukocytes using a DNA extraction kit (Taingen Biotech, China). After DNA extraction, reactions were carried out in a final volume of 20 μL containing 100 ng DNA, 2.5 mM MgCl2, 1.25 units of Taq DNA polymerase, 1 μL of each primer, 250 μL dNTPs mixture, and 1X PCR buffer. All PCR reaction reagents were purchased from Fermentas (Lithuania). Genotyping for the G/A polymorphism was carried out using polymerase chain reaction with sequence-specific primers (PCR-SSP). The following primers were used to amplify the target fragments: for *COMT-A*, 5'-TggTggATTTCgCTggCA-3' (forward) and 5'-ACACC CATACAAgcaTTCATCAgTT-3' (reverse); for *COMT-G*, 5'-gCATgCACACCTTgTCCTTCAC-3' (forward) and 5'-TgAgCATAgAggCTAAgggACCAT-3' (reverse). The PCR conditions were as follows: preheating for 1 min at 96 °C to achieve a hot start, then a touch-down (TD) procedure consisting of denaturation at 96 °C for 20 s and annealing at 70 °C for 45 s during the first five cycles, followed by 21 cycles of 96 °C for 25 s, 65 °C for 50 s, and 72 °C for 30 s each, then 21 cycles at 96 °C for 30 s, 55 °C for 60 s, and 72 °C for 90 s, and a final extension at 20 °C for 2 min.

The size of the amplified PCR products was 455 bp for the *COMT-158 G* allele and 322 bp for the *COMT-158 A* allele. These products were analyzed by 2% agarose gel electrophoresis and visualized with 0.5 μg/mL ethidium bromide staining under an ultraviolet illuminator. Genotypes were scored by two independent individuals, and any ambiguous genotypes were repeated or omitted. PCR products were identified by sequencing.

Chi-square tests were used to evaluate the differences in the frequency distributions of selected demographic variables between the cases and controls, including each allele and genotype of the *COMT* polymorphisms. Unconditional univariate and multivariate logistic regression analyses were performed to obtain the crude and adjusted odds ratios (ORs) for the risk of psoriasis and their 95% confidence intervals (CIs). The multivariate adjustment included age and gender variables. Two-tailed tests of statistical significance were performed with SAS software (version 8.2; SAS Institute, Inc., Cary, North Carolina).

We found no significant differences in age and gender between the psoriasis patients and the control group: mean age was 32.1 ± 13.6 years in the psoriasis group and 31.5 ± 13.9 years in the control group (p = 0.232), while the gender distributions (M:F) were 54.8%:45.2% in the study group and 50.5%:49.5% in the control group (p = 0.157) (Table 1).

The genotype and allele frequencies of *COMT158* in the study and control groups are shown in Table 2. In the control group, the genotype frequencies of the *COMT 158* polymorphism were in agreement with the Hardy-Weinberg equilibrium (p = 0.168). No significant differences were found between the psoriasis and control subjects regarding the frequencies of genotypes *GG*, *GA*, and *AA* (p = 0.759) (Table 2). Similarly, there was no significant difference between the two groups regarding the frequencies of allele *A* (p = 0.624).

To consider the single nucleotide polymorphism in codon 158 (*G* > *A*) of the *COMT* gene, which leads to a valine-methionine substitution resulting in the difference in *COMT* activity, we analyzed the association between combined genotypes (*GG, GA+AA*) and the risk of psoriasis vulgaris. We investigated whether the distributions of combined genotypes (*GG, GA+AA*) were different among type 1 and type 2 psoriasis patients and control subjects. However, as shown in Table 3, the frequency of the variant combined genotype (*GA+AA*) was not statistically different in the type 1 psoriasis (43.5%) and type 2 psoriasis (47.8%) cases compared to controls (43.0%).

The Psoriasis Area and Severity Index (PASI) is a widely used method to characterize the severity of the disease (de Rie *et al.*, 2004). In our study, PASI was used to classify the psoriatic patients into two different levels: level 1 = PASI ≤ 20; level 2 = PASI > 20. As shown in Table 3, the frequency of the variant combined genotype (*GA+AA*) was 45.5% in level 1 and 41.5% in level 2 psoriasis patients. There was no statistical difference between the two levels of psoriasis and the controls.

Based on previous research, we hypothesized that polymorphism *COMT158 (G* > *A)* might be associated with the risk of psoriasis vulgaris. Our study, however, found no significant association between *COMT158 (G* > *A)* and psoriasis vulgaris, and also no significant difference between *COMT158 (G* > *A)* and different types of psoriasis vulgaris. Our study included only statistical research, not functional investigation of the association between the *COMT* polymorphism and psoriasis vulgaris, so this hypothesis needs to be tested by further functional studies.

In conclusion, we found no significant difference regarding the *COMT-158 (G* > *A)* polymorphism between psoriasis vulgaris patients and control subjects in a Han Chinese population. The difference between these results and those of Erdal *et al.* (2004) may be due to differences in the ethnic composition of the populations and in the number of investigated subjects. Our findings suggest that polymorphism *COMT 158* may not be associated with the risk of psoriasis vulgaris. Larger population-based studies, different clinical subgroups and studies among different ethnic groups are needed to confirm these findings.

## Figures and Tables

**Table 1:** Frequency distributions of selected variables in the psoriasis cases and psoriasis-free controls.

Variables	Cases (n = 524)			Controls (n = 549)		p^a^
	n	%		n	%	
Age (years)						0.232
≤ 10	18	3.4		22	4.0	
11-20	102	19.5		136	24.8	
21-30	138	26.3		140	25.5	
31-40	133	25.4		114	20.8	
41-50	83	15.8		79	14.4	
> 51	50	9.5		58	10.6	

Gender						0.157
Male	287	54.8		277	50.5	
Female	237	45.2		272	49.5	

^a^Two-sided χ^2^ test for the frequency distributions of selected variables between the cases and controls.

**Table 2:** Genotype and allele frequencies of polymorphism *COMT* and associations with psoriasis risk.

Genotype	Cases (N = 524)			Controls (N = 549)^a^		p^b^	Crude OR (95% CI)	Adjusted OR (95% CI)^c^
	N	%		N	%			
GG	294	56.1		313	57.0		1.00	1.00
GA	201	38.4		211	38.4	0.759	1.02 (0.79-1.31)	1.00 (0.78-1.29)
AA	29	5.5		25	4.6		1.24 (0.71-2.16)	1.23 (0.70-2.15)
GA+AA	230	43.9		236	43.0	0.765	1.04 (0.82-1.33)	1.02 (0.80-1.31)
A allele	0.247			0.237		0.624		

^a^The genotype frequencies observed in the control subjects were in agreement with the Hardy-Weinberg equilibrium (χ^2^ = 1.900, p = 0.168). ^b^Two-tailed χ^2^ test for either genotype distributions or allele frequencies between the cases and controls. ^c^Odds ratios (ORs) were obtained from a logistic regression model with adjustment for age and gender; 95% confidence interval (CI).

**Table 3:** Association and stratification analyses of the combined genotypes of polymorphism *COMT* and risk of psoriasis.

Variables	N (case/control)	Combined genotypes (case/control^a^)					Crude OR (95% CI)	Adjusted OR (95% CI) ^b^	p^c^
		GG			GA+AA				
		n	%		n	%			
Total	524/549	294/313	56.1/57.0		230/236	43.9/43.0	1.04 (0.82-1.33)	1.02 (0.80-1.31)	0.765
Onset age									
≤ 40	478/549	270/313	56.5/53.7		208/236	43.5/43.0	1.02 (0.80-1.31)	1.03 (0.80-1.32)	0.865
> 40	46/549	24/313	52.2/57.0		22/236	47.8/43.0	1.22 (0.67-2.22)	0.81 (0.40-1.63)	0.525

PASI									
≤ 20	317/549	173/294	54.6/53.7		144/254	45.5/46.4	1.11 (0.84-1.47)	1.09 (0.82-1.44)	0.468
> 20	209/549	122/313	58.4/57.0		87/236	41.5/46.4	0.95 (0.69-1.31)	0.94 (0.68-1.30)	0.735

^a^The observed genotype frequencies of the controls were in agreement with the Hardy-Weinberg equilibrium (χ^2^ = 1.900, p = 0.168). ^b^Odds ratios (ORs) were obtained from a logistic regression model with adjustment for age and gender; 95% confidence interval (CI). ^c^Two-tailed χ^2^ test for either genotype distributions or allele frequencies between cases and controls.
